# Epigenetics Meets Radiation Biology as a New Approach in Cancer Treatment

**DOI:** 10.3390/ijms140715059

**Published:** 2013-07-18

**Authors:** Joong-Gook Kim, Moon-Taek Park, Kyu Heo, Kwang-Mo Yang, Joo Mi Yi

**Affiliations:** 1 Research Center, Dongnam Institute of Radiological & Medical Sciences (DIRAMS), Busan 619-953, Korea; E-Mails: jgkim210@gmail.com (J.-G.K.); krishuna73@gmail.com (M.-T.P.); khjhk33@gmail.com (K.H.); kmyang@dirams.re.kr (K.-M.Y.); 2 Radiological Oncology, Dongnam Institute of Radiological & Medical Sciences (DIRAMS), Busan 619-953, Korea

**Keywords:** epigenetic regulation, DNA methylation, histone modification, radiation exposure, cancer, colon cancer

## Abstract

Cancer is a disease that results from both genetic and epigenetic changes. In recent decades, a number of people have investigated the disparities in gene expression resulting from variable DNA methylation alteration and chromatin structure modification in response to the environment. Especially, colon cancer is a great model system for investigating the epigenetic mechanism for aberrant gene expression alteration. Ionizing radiation (IR) could affect a variety of processes within exposed cells and, in particular, cause changes in gene expression, disruption of cell cycle arrest, and apoptotic cell death. Even though there is growing evidence on the importance of epigenetics and biological processes induced by radiation exposure in various cancer types including colon cancer, specific epigenetic alterations induced by radiation at the molecular level are incompletely defined. This review focuses on discussing possible IR-mediated changes of DNA methylation and histone modification in cancer.

## 1. Introduction

Epigenetic alterations are heritable changes in the structure and function of the genome that occur without changes in the DNA sequence. In mammalian cells, these epigenetic changes consist primarily of DNA methylation and post-translational histone modifications. Both types of modifications have been shown to play critical biological roles, *i.e.*, in normal growth, development, and differentiation in multiple organisms [1]. Epigenetic modifications are defined as heritable information other than nucleotide sequences. It is well known that aberrant epigenetic mechanisms manifest in both global changes in chromatin packaging and in localized gene promoter changes that influence the transcription of genes involved in cancer development [2]. Epigenetic regulation has recently been established as an emerging mechanism of cancer therapy. Therefore, the understanding of the epigenetic mechanism in cancer is required for the development of epigenetic therapies related to critical biological aspect.

The central paradigm of classical radiation biology is based on a model that considers only the direct interaction of ionizing radiation (IR) with the genetic material in the nucleus. This interaction could have an effect on several biological aspects in IR-exposed cells. IR is now generally accepted as a severe DNA-damaging agent, which can lead to severe diseases such as cancer [3]. It has been also well known that the effects of IR on the genomic instability, *i.e.*, gross chromosomal aberrations, deletions, insertions, and point mutations, have a transgenerational nature and, thus, are considered to be the precursors of tumorigenesis and genetic effects [4]. The point of view of the transgenerational nature of genomic instability suggests the possible involvement of epigenetic mechanisms.

## 2. Epigenetic Regulation in Cancer

Cancer research in epigenetics in the 1990s was dominated by a focus on understanding and extending the discoveries of DNA methylation abnormalities [5]. In the past 15 years, with an explosion of knowledge, this focus has integrated the role of histone codes from chromatin modifications and organization and their relevance to gene expression [6]. In an emerging view, these results may now be called “the cancer epigenome”, which harbors numbers of abnormalities that are based on somatically heritable alterations that are not due to primary DNA sequence changes [2]. The fundamental of the impact of epigenetic changes in cancer depends on pinpointing at what stages of cancer development and how they influence the biology of each progression step towards the invasive disease. While epigenetic changes, e.g., genetic alterations, may arise at any step, it is increasingly apparent that many chromatin-mediated abnormalities appear before invasive cancer has developed [2]. There are two primary and interconnected epigenetic mechanisms, *i.e.*, DNA methylation and histone modification [7,8].

DNA methylation plays a central role in the epigenetic control of genomic programs in both normal and cancer cells [9]. In mammalian genomic DNA, methylation occurs on the fifth carbon position of cytosine bases that are part of CpG dinucleotides. CpG dinucleotides have been progressively depleted from the eukaryotic genome over the course of evolution [10]. The remaining CpG dinucleotides in the mammalian genome are often methylated. CpG dinucleotides are not uniformly distributed throughout the human genome. Rather, they are concentrated in specific regions (CpG islands) that are located in the upstream region from the transcriptional start site of many genes as well as at other regions. In human, approximately 60% of all genes have CpG islands, of which the majority is unmethylated in all normal tissue types and throughout development, which was demonstrated by using computational analyses [11]. Methylation of these CpG islands is a primary method by which stable, heritable gene silencing is achieved. However, in human cancers, many significant changes in DNA methylation patterns occur during oncogenesis and tumor progression. These changes can be either broad changes that involve large regions of DNA or locus-specific changes that control the transcription of specific genes. One of the first epigenetic alterations noted in tumors was global hypomethylation. Hypomethylation occurs broadly throughout the DNA of cancer cells, affecting repetitive sequences, intergenic areas, and promoter CpG islands associated with genes [12].

The most well studied abnormality of DNA methylation in cancer is hypermethylation, which is now established as a very specific event in cancer cells and often involves normally unmethylated gene promoter CpG islands [9]. This promoter change can be associated with transcriptional silencing and, thus, loss of function of tumor suppressor genes and may be a key event contributing to the oncogenic process [2,13,14]. This method of tumor suppressor inactivation is one of the most prevalent modes in cancer and is found in nearly every type of human malignancy [2,15]. Tumor suppressors that are inactivated by hypermethylation can affect DNA repair, programmed cell death, angiogenesis, cell cycle regulation, and tumor cell invasion. Various tumor suppressor genes, including retinoblastoma (*Rb*), *CDKN2A* (*p16*), *hMLH1*, and *VHL*, are tumor-specifically silenced by CpG island hypermethylation of their promoters [9,16]. Recently, many studies reported DNA methylation as a biomarker for the early detection of cancer and as a tool for monitoring patients with different types of cancer. In particular, in colorectal cancer, there are comprehensive studies on the biological significance of tumor suppressor genes as well as identification of methylation biomarkers for clinical use. Methylated genes that have been well characterized in colon cancer are described in Table 1.

## 3. Radiation Biology in Cancer

Ionizing radiation (IR)-induced carcinogenesis was the first biological event observed immediately after the discovery of X-rays. IR can damage cellular components, including proteins, lipids, and nucleic acids. In particular, IR has been known to induce DNA double-strand breaks (DSBs) in cells [29]. Incorrect repair or accumulation of DSBs in irradiated cells can lead to cell cycle arrest and multiple unbalanced chromosomal rearrangements [4]. Moreover, incomplete cell cycle arrest and chromosomal aberrations have been known to induce aneuploidy, which is a typical characteristic of cancer [4]. Although IR is, thus, a well-known carcinogenic agent, it has been commonly applied as cancer therapeutic tool, to date [3,4,30,31], because IR effectively induces cell death in rapidly dividing cells, e.g., cancer cells [32]. Therefore, the clonogenic death of cancer cells via direct effects of IR is highly significant as response of tumors to radiotherapy. In addition, a growing body of evidence revealed that radiotherapy can be also critically affected by the tumor microenvironment, including vasculatures [33]. The sensitivity of malignant cancer cells to IR is highly influenced by the supply of oxygen via the circulating blood system [33]. Thus, conventional fractionated radiotherapy is widely used in cancer patients because it causes relatively little damage in tumor vasculatures, as well as surrounding normal tissues, thereby allowing reoxygenation of hypoxic cancer cells [34]. On the other hand, hypofractionated radiotherapy such as stereotactic body radiation therapy or stereotactic radiation surgery has been shown to cause severe damage leading to destruction of the both tumor microenvironment and cancer cells [34]. In this notion, it was suggested that radiotherapy-induced tumor regression results from apoptotic death of endothelial cells comprising the tumor vasculature [35]. However, this implication was controverted by those who believe that the immediate cause of radiation-induced tumor regression is its direct influence on cancer cells [36].

Although radiotherapy is recognized as one of the major cancer treatment modalities, cancers have been known to show a propensity for recurrence after radiotherapy [37]. In this context, a decisive subcellular population of radiation-resistant cancer cells with potent tumorigenic activity is responsible for cancer relapse. In addition, emerging evidences indicated that such a subpopulation of cancer cells possessing stem cell properties is responsible for tumor initiation, maintenance, and progression [38]. These cancer-initiating cells have been named cancer stem cells and characterized by their potent tumorigenic properties and ability to self-renewal [39]. More importantly, the cancer stem cell population also contributes to resistance to radiotherapy, as well as chemotherapy, and is believed to mediate cancer relapse after anti-cancer treatments [40]. Furthermore, cancer stem cell markers have been reported to be epigenetically regulated [41,42]. For instance, Bmi1, a cancer stem cell marker in colon, breast, and brain tumors, is known as a key regulatory subunit of the polycomb repressive complex-1, which was shown to epigenetically maintain stemness of cancer stem cells and suppress terminal differentiation through Hedgehog signaling pathways [43]. Further investigations are still needed to define the potential involvement of epigenetic alterations in cancer stem cells, which might provide important clues for improving the efficacy of radiation therapy.

## 4. Epigenetic Regulation and Radiation

### 4.1. DNA Methylation and Radiation

DNA methylation is one of the most common mechanisms of epigenetic regulation. As mentioned above, DNA methylation was well studied in many different types of cancer model systems such as colon, breast, lung, and prostate cancer with abnormal epigenetic alterations [5,12]. Two types of alteration in the DNA methylation pattern occur in cancer, *i.e.*, hypo- and hypermethylation of specific genes, as well as global genome DNA methylation changes [44]. Most research in the field of cancer epigenetics has been focused on the role of hypermethylation of the promoters of tumor suppressor genes, as well as their clinical relevance [5]. However, there are much fewer studies of DNA hypomethylation in human diseases, including cancer, even though the global DNA hypomethylation pattern is a well-known characteristic of cancer cells. This occurs specifically to mobile genetic elements such as repeat elements, *LINE1*, *Alu*, *etc.* [45].

**Figure 1:**
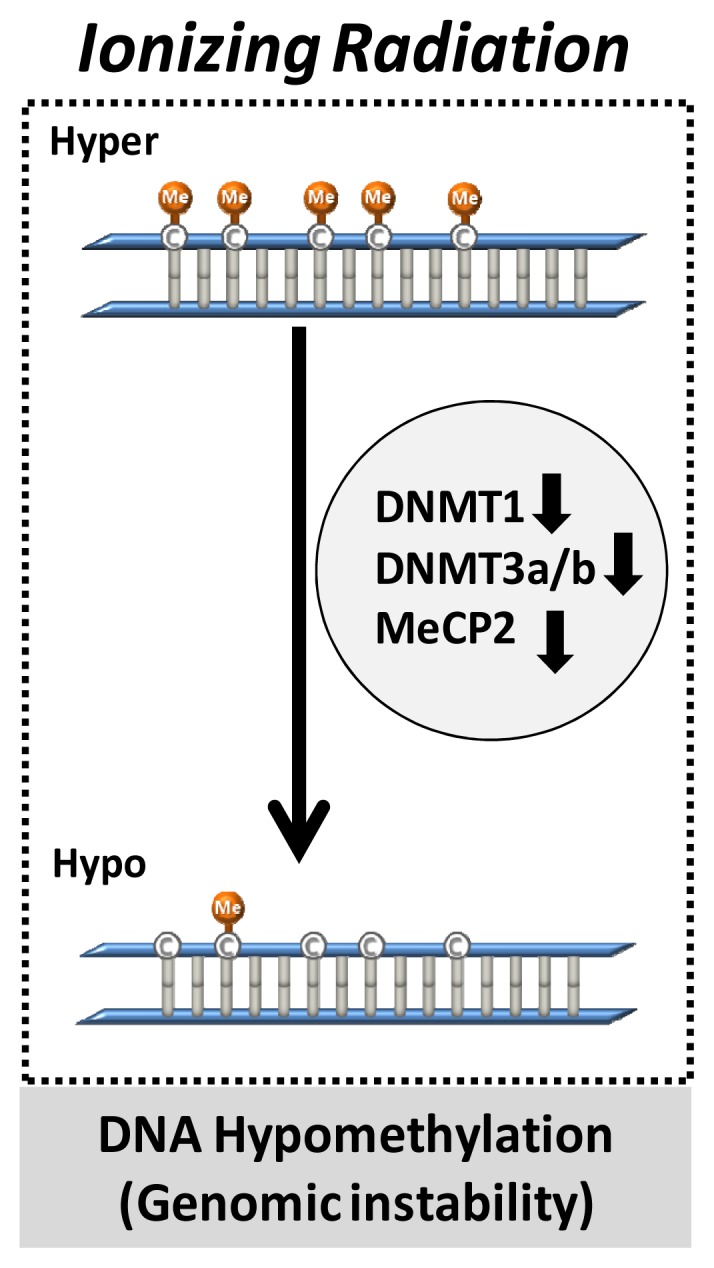
Radiation effect on DNA methylation. The schematic shows the change of global DNA methylation by radiation exposure in a cancer system. Radiation might induce global DNA hypomethylation through a decrease in DNA methyltransferases, including DNMT1, DNMT3a, DNMT 3b, and MeCP2. This phenomenon results in genomic instability in cancer.

At present, there are several reports demonstrating that IR exposure could affect DNA methylation patterns. IR exposure has been found to have dose-dependent, sex, and tissue-specific effects on global hypomethylation using mouse model system [46]. Mostly hypomethylation, loss of methylation, paralleled with a decrease in the DNA expression levels of methyltransferases (DNMTs; DNMT1, DNMY3a, and DNMT3b) and methyl CpG binding proteins (MeCP2) was associated with radiation-induced changes [47,48]. These results suggest that DNA hypomethylation patterns induced by radiation exposure could result in genomic instability in the exposed animals (Figure 1).

Little is known about how DNA methylation alters in cancer cells after radiation exposure. There are interesting reports on the relationship between a DNA methyltransferase inhibitor (5-azacytidine) and radiation sensitivity in colon cancer. Hofstetter *et al.*, 2010 and Cho *et al.*, 2009 have reported that genomic hypomethylation induced by 5-azacytidine results in enhanced radiation sensitivity in colon cancer [49,50]. These results still leave many questions unanswered, *i.e.*, defining the specific factors associated with radiation sensitization after loss of DNA methylation that might be a useful target for radiation sensitization. In addition, Kuhmann and colleagues [51] demonstrated that breast cancer cells treated with fractionated IR showed several locus-specific DNA methylation alterations, which were mostly loss of methylation (*TRAPP9*, *FOXC1*, and *LINE1*). More recently, it has been reported that there are differential DNA methylation changes between radiosensitive *vs.* radioresistant cancer cells [52].

Even though cancer research using radiation exposure has been very well characterized by many researchers, the underlying epigenetic mechanisms that drive alterations, in particular, site-specific changes in DNA methylation patterns remain elusive. Further comprehensive studies on radiation-induced epigenetic alterations in cancer may open a new avenue for developing cancer targeting therapies.

### 4.2. Histone Modification and Radiation

Positioning of the nucleosome with its 147 base pairs of DNA wrapped around the octamer of the core histones, H2A, H2B, H3, and H4, in conjunction with the above modifications of histones, modulates the normal epigenome in terms of maintaining gene expression patterns and normal chromosome structure and function [6]. To understand the origins of epigenetic alterations in cancer, we must use the increasing knowledge about molecular control for organizing and maintaining the chromatin structure of the normal nucleus and how histone modifications, including lysine acetylation, lysine and arginine methylation, serine and threonine phosphorylation, glutamic acid ADP-ribosylation, and lysine ubiquitination and sumoylation participate in this process [6] (Table 2). Cancers have not only altered DNA methylation but also caused global changes in the levels of proteins that participate in chromatin modifications, e.g., polycomb complex components, and in histone modifications, e.g., acetylation and methylation of lysine residues on histones H3 and H4 [53,54].

The strong dependence between DNA methylation and chromatin modifications for DNA packaging is known. Furthermore, histone modification and DNA methylation closely interact in the setting of the transcriptional states of chromatin. Especially in cancer, silenced genes regulated by DNA hypermethylation can be models to examine the chromatin control of gene expression. When such genes are expressed with no methylation, their promoters have virtually identical distribution of the active marks, H3K9acetyl and H3K4me [55,56]. In contrast, when silenced genes are associated with hypermethylation, the distribution of these active marks is severely decreased, and virtually every histone methylation mark, including mono-, di-, and trimethylation of H3K9 and H3K27 that has been associated with transcriptional repression, is enriched [56].

**Figure 2:**
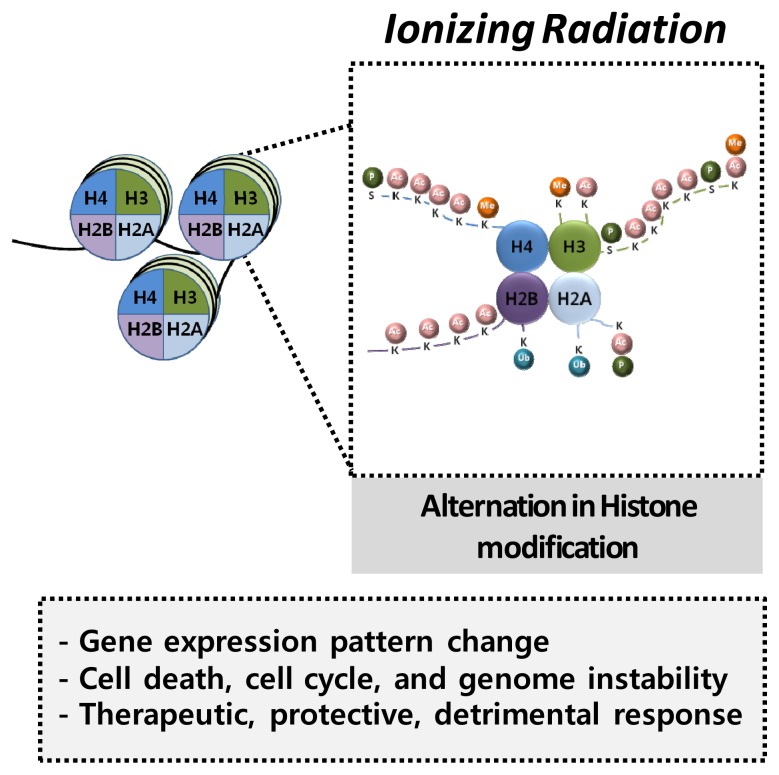
Radiation effect on histones. Radiation can induce phosphorylation of histone H2AX, and trimethylation of histone H4K20, which affect gene expression patterns, consequently leading to cell death, changes in cell cycle, and genomic instability. These events may be closely related to the therapeutic, protective, or detrimental response to irradiation.

Radiation-induced phosphorylation of γH2AX was extensively studied as a measure of DSB accumulation in irradiated cells [74]. Regarding IR exposure, phosphorylation of histone H2AX at serine 139 (γH2AX) is well studied as histone modification affected by IR exposure because γH2AX is one of the first signals for a cellular response to DSB, as well as IR exposure [75,76]. γH2AX accumulates in the nucleus at DSBs forming the γH2AX loci, and a direct correlation has been found between γH2AX phosphorylation and the number of DSBs resulting from radiation [77]. Therefore, γH2AX is crucially important for the repair of DNA strand breaks and for the maintenance of genome stability [77] (Figure 2).

Pogribny *et al.* [78] suggested that X-ray irradiation in a mouse model induced a decrease in trimethylation of histone H4K20 in the thymus and, eventually, could result in an overall relaxation of the chromatin organization. In addition, gamma-irradiation could result in relaxation of the chromatin structure around the DSB immediately after exposure. However, after some time, methylation of H3K9 increased and chromatin obtained the condensed state [79]. The influence of radiation exposure on histone modification as variable epigenetic mechanism is almost unexplored so far.

### 4.3. Small RNAs and Radiation

Epigenetic regulation can be controlled by small RNAs such as microRNA (miRNA). MicroRNAs (miRNAs) are short, 18–25-nucleotide long, non-coding single-stranded RNAs, which are capable to regulate gene expression on post-transcriptional level through binding to their target protein-encoding mRNAs [80]. It is estimated that more than 1000 miRNAs are transcribed and that 30% of the human genome is under miRNA regulation, one miRNA being able to modulate post-transcriptionally hundreds of down-steam genes. Regarding this, miRNA controls a wide range of biological process including cellular differentiation, proliferation, apoptosis, and stem cell maintenance [80]. Recently, miRNAs can act as tumor suppressors when their loss of function can initiate or contribute to the malignant transformation of a normal cell. The loss of function of a miRNA could be due to several mechanisms including genomic deletion, mutation, epigenetic silencing, and miRNA processing alterations [81–83]. Several miRNAs that have been described to have tumor suppressive function in colon cancer are miR-195 [84], miR-143 [85], miR-34a [86], and miR-133 [87].

miRNAs are also involved in IR-induced response *in vitro* and *in vivo*. Studies on the effects of IR exposed whole body rodent cause altered miRNA expression pattern in tissue specific as well as sex-specific protective mechanism [88]. In addition, altered expression pattern of miRNA have also been profiled in directly exposed males, as well as their unexposed offspring, demonstrating the possibility that they may also play a role in transgenerational epigenetic inheritance of genome instability [89].

## 5. Colon Cancer: Radiation and Epigenetics

Colon cancer is a very well characterized cancer type, which accumulates genetic and epigenetic alterations that transform normal colonic epithelial cells to adenocarcinoma cells. Epigenetic alterations play a major role in the initiation and progression of colorectal cancers (CRCs); epigenetic instability appears to be a common phenomenon in CRC. Unlike genetic mutations, epigenetic alterations are reversible. This phenomenon established the development of DNMT inhibitors as a clinical concept for cancer therapy. The cytosine analogues 5-azacytidine (azacytidine) and 2′-deoxy-5-azacytidine (decitabine) are pharmacological inhibitors of DNMT and are currently being developed as drugs for epigenetic cancer therapy [90]. Although DNMT inhibitors are not currently used in CRC, both drugs have received Food and Drug Administration (FDA) approval for the treatment of myelodysplastic syndrome, a pre-leukemic bone marrow disease [91,92].

For colon cancer, surgery is the main treatment for early stage. After surgery, it can be used in combination with mostly chemotherapy or radiation therapy in some case. However, role of radiation in colorectal cancer is not well-defined yet. In addition, radiation therapy is not a common way to treat colon cancer, though it may be used in certain situations. Radiation therapy, often with chemotherapy, is frequently used in the adjuvant or nonadjuvant setting for the treatment of rectal cancers, whereas chemotherapy alone is more common for the adjuvant and neoadjuvant treatment of colon cancers [93].

With regard to radiation therapy, many of the common, moderately radioresponsive tumors are routinely treated with curative doses of radiation therapy if they are at an early stage, e.g., head and neck cancer, breast cancer, non-small cell lung cancer, cervical cancer, prostate cancer, and, recently, rectal cancer. Since radiation therapy is a local treatment, there are a few in whole addition of local or regional radiation may improve control of the disease and help the patient’s survival. In recent years, adjuvant radiotherapy with or without chemotherapy has been widely adopted to improve oncologic outcomes in patients with rectal cancer treated by conventional blunt surgery [94].

Epigenetic alterations, especially aberrant DNA methylation, are becoming increasingly recognized as a causal mechanism in CRC. Histone modifications accompany DNA hypermethylation changes in CRC and likely establish a complex network to maintain gene silencing. Continued investigations into the role of epigenetic mechanisms and radiation biology in CRC are likely to yield important information on new potential targets for therapy.

## 6. Conclusions

The molecular mechanisms contributing to the etiology of radiation-induced cancer consist of prominent genetic and epigenetic changes that have been the subject of extensive research in recent years. Epigenetic changes have become increasingly recognized as important factors contributing to cancer development. Epigenetic approaches (DNA methylation and histone modification) could be a critical research field to understand the biological effects of IR in cancer.

## Figures and Tables

**Table 1:** Genes are regulated by DNA methylation from colorectal cancers.

Gene	Biological function	References
*RASSF1A*, *CDKN2A*, *CHFR*, *DLEC1*, *MYOD*, *RGC-32*	cell cycle arrest	Lee *et al.*, 2009 [17]; Borinstein *et al*., 2010 [18]
*MGMT*, *hMLH1*, *hMLH2*	DNA repair	Lee *et al.*, 2009 [17]
*APC*, *SFRP1*	Wnt pathway	Lee *et al.*, 2009 [17]
*RUNX3*, *TIMP3*, *DCC*	apoptosis	Nishio *et al.*, 2010 [19]
*CDH13*, *ADAM23*	cell to cell interaction	Wang *et al.*, 2011 [20]
*MINT*	Notch pathway	McGivern *et al.*, 2004 [21]
*UNC5C*, *DCC*, *EVL*, *VIM*, *FLNC*	cytoskeleton remodeling and cell polarity	Bernet *et al*., 2007 [22]
*COX2*	inflammation	Asting *et al.*, 2011 [23]
*CDH1*	invasion and metastasis	Graziano *et al.*, 2004 [24]
*HLTF*	chromatin remodeling factor	Moinova *et al.*, 2002 [25]
*RAR-b*, *SMAD4*, *TWIST1*	growth and differentiation	Isaksson *et al.*, 2012 [26]
*Wif-1*	mesoderm segmentation	Lee *et al.*, 2009 [17]
*SOCS1*, *SEPT9*	cytokine signaling	Grützmann *et al.*, 2008 [27]; deVos *et al.*, 2009 [28]

**Table 2:** Histone modification changes in multiple cancers.

Cancer type	Histone modification changes	References
		
Lung	H4K16ac, H3K18ac, H4K8ac, H4K5ac, H3K9ac, H4K12ac, H4K16ac	Song *et al.*, 2012 [57]; Barlési *et al.*, 2007 [58]; Seligson *et al.*, 2009 [59]

	H3K4me2, H3K9me3, H4K20me3	
		
Prostate	H3Ac, H4Ac, H3K18ac	Ellinger *et al*., 2010 [60]; Seligson *et al.*, 2009 [59]; Behbahani *et al.*, 2012 [61]; Bianoco-Miotto *et al.*, 2010 [62]

	H3K4me1, H3K9me2, H3K9me3, H3K4me2, H3K27me3, H4K20me1	
		
Breast	H3K18ac, H4K12ac,, H4K16ac	Elsheikh *et al*., 2009 [63]; Leszinski *et al.*, 2012 [64]

	H3K4me2, H3K9me3, H4K20me2, H4K20me3, H4R3me2	
		
Leukemia	H3K9me3	Muller-Tidow *et al.*, 2010 [65]
		
Stomach	H3K9me3, H3K27me3	Park *et al.*, 2008 [66]; Zhang *et al.*, 2009 [67]
		
Esophagus	H3K18ac	Tzao *et al.*, 2009 [68]; Cohen *et al.*, 2011 [69]

	H4R3me2,H3K27me3, H4R3me2	
		
Kidney	H3K4me1, H3K4me2, H3K4me3, H3K9me1, H3K27me1, H3K27me2, H3K27me3	Ellinger *et al.*, 2010 [60]; Rogenhofer *et al.*, 2012 [70]
		
Liver	H3K4me3, H3K27me3	He *et al.*, 2012 [71]; Cai *et al.*, 2011 [72]
		
Pancreas	H3K4me2, H3K9me2, H3K18ac	Manuyakorn *et al.*, 2010 [73]

colon	H3K4me2, H3K9ac, H3K9me2, H3K27me3	Seligson *et al.*, 2009 [59]
